# Multiple Endocrine Neoplasia 2a Presenting with Pheochromocytoma and Pituitary Macroadenoma

**DOI:** 10.5402/2011/732452

**Published:** 2011-04-18

**Authors:** Jonathan E. Heinlen, David D. Buethe, Daniel J. Culkin, Gennady Slobodov

**Affiliations:** Department of Urology, University of Oklahoma Health Sciences Center, 920 Stanton L. Young Boulevard, WP 3150, OK 73104-5036, USA

## Abstract

Multiple Endocrine Neoplasia type 2A (MEN-2a) is a rare disease associated with tumors of endocrine organs. Presentation most commonly is with medullary thyroid cancer and infrequently with other complaints. Pituitary adenoma has been seen coincidentally with this disease very rarely. Presented is a case of coincident MEN-2a with a symptomatic pituitary adenoma and an asymptomatic pheochromocytoma. A brief review is also provided.

## 1. Introduction

Pheochromocytoma is a physiologically active tumor of the adrenal gland that occurs in isolation or in association with Multiple Endocrine Neoplasia type 2a (MEN-2a). Rarely is pheochromocytoma the presenting disease process in MEN-2a and even more rarely is it associated with pituitary macroadenoma. We present a case of MEN-2a presenting in a highly unusual manner that brings into question the need for more extensive workup prior to treatment for pheochromocytoma.

## 2. Case Presentation

**Figure 1:**
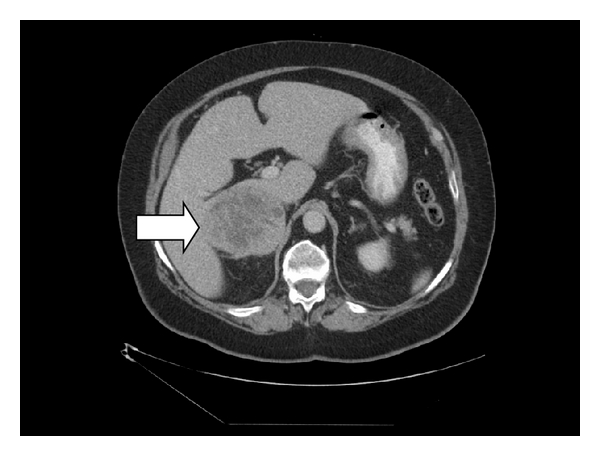
CT of the abdomen showing an adrenal mass adherent to the IVC.

**Figure 2:**
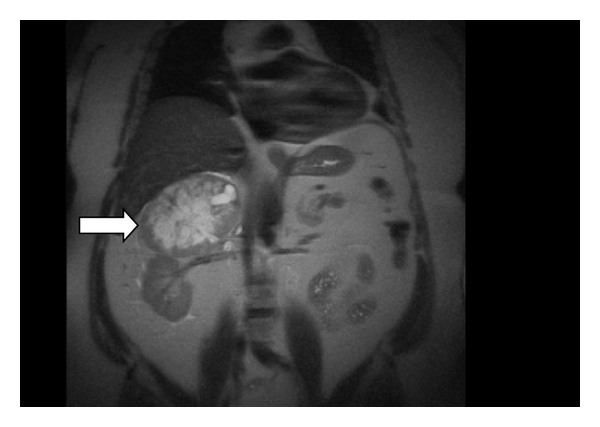
T2-Weighted MRI of the abdomen demonstrating high-attenuation characteristic of pheochromocytoma.

A 62-year-old white male presented with right abdominal and flank pain of gradual onset over several months. He had a history of hypertension controlled with atenolol only and had no history of paroxysmal hypertensive episodes. Workup revealed a 13 cm right adrenal mass by CT (Figure 1) which on T2-weighted MRI was high attenuating in nature (Figure 2). Due to suspicion for pheochromocytoma, serum and urinary metanephrines were obtained. Serum Normetanephrine was 7086 *μ*g/mL and serum metanephrine was 538 *μ*g/mL. Urinary Normetanephrine was 29,653 *μ*g/mL (normal 75–375 *μ*g/mL) and metanepherine was 2560 *μ*g/mL (normal 24–96 *μ*g/mL). Prior to planned right open adrenalectomy, blockade was begun with 10 mg daily of phenoxybenzamine. 

Open adrenalectomy was complicated with intraoperative hypertensive crisis, difficult exposure due to the size of the tumor, and ultimately right nephrectomy in order to facilitate removal of the tumor. Adherence to the inferior vena cava (IVC) necessitated partial resection of the IVC with primary closure. Once the tumor vasculature was isolated, hypotension ensued requiring pressor support. The patient remained intubated postoperatively and was extubated on postoperative day one. Later the same day, the patient complained of headache and left sided visual field loss. Exam confirmed left lateral hemianopsia. These findings were not present preoperatively based on history and physical exam.

**Figure 3:**
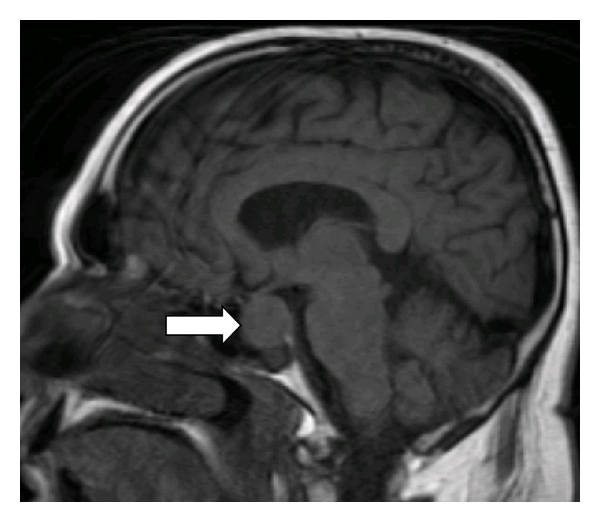
MRI of the brain showing pituitary macroadenoma compressing the optic chiasm.

MRI of the head was performed to evaluate visual field defect and a 1 cm pituitary macroadenoma was found compressing the optic chiasm (Figure 3). At this point full endocrine evaluation was obtained including all pituitary hormones, thyroid hormones, parathyroid hormone, and calcitonin. Also evaluation of the RET proto-oncogene demonstrated a missense mutation (*C618S*) in exon 10 of the gene. Parathyroid hormone was elevated at 155 pg/mL and calcitonin was slightly above normal at 116 pg/mL. Sonogram of the thyroid demonstrated bilateral involvement of the thyroid with cystic nodules. Fine needle aspiration (FNA) suggested medullary thyroid carcinoma and so thyroidectomy was performed along with subtotal parathyroidectomy.

**Figure 4:**
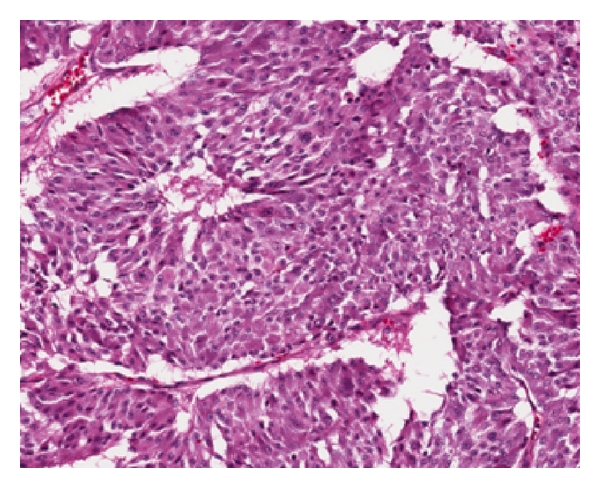


Pathology of the adrenal specimen demonstrated pheochromocytoma with focal capsular penetration but tumor-free margins (Figure 4). Thyroid pathology was bilateral medullary thyroid carcinoma pT1 with no clinical nodes or metastases. The pituitary adenoma was treated with transsphenoidal resection, however the patient did not regain visual function in followup. At 12 months followup he remains tumor-free and normotensive on atenolol.

## 3. Discussion of Diagnosis

Multiple Endocrine Neoplasia syndrome type 2a (MEN-2a) is an autosomal dominant hereditary syndrome caused by mutation of the tyrosine kinase RET proto-oncogene on chromosome 10q11.2. The hallmark of the disease is medullary thyroid cancer (MTC) which occurs in nearly 100% of cases [1]. Pheochromocytoma is identified in 30–50% of patients with MEN-2a. Hyperparathyroidism occurs in 20% of patients and is usually mild and manifests as only a slight elevation of serum calcium [2]. Most cases present with MTC primarily and through screening and followup are found to have other associated tumors. Only 15% of MEN-2a patient present with pheochromocytoma as the primary complaint and 25% have concomitant MTC and pheochromocytoma. Most commonly pheochromocytoma is discovered at a mean of 10 years after the MTC is discovered and treated [1]. 

Treatment of MTC is typically total thyroidectomy with bilateral neck dissection for lymphadenectomy. Screening for pheochromocytoma is performed yearly with urinary metanephrine assessment. Unilateral pheochromocytoma is commonly amenable to laparoscopic resection [3]. Partial adrenalectomy for small tumors has been described and has a similar complication rate to total adrenalectomy. This approach may be considered in patients with MEN-2a due to their recurrence rate of 10–20% [1, 4]. Management of hyperparathyroidism depends on the patient's preoperative calcium and parathyroid hormone levels. If the patient is eucalcemic, then only removal of obviously enlarged parathyroid glands should be done and otherwise the parathyroid should be spared [5].

Surgery for large pheochromocytoma can be a difficult and dangerous task. Small series have shown little difference in complication rate between laparoscopic and open approach, however the risk of open conversion due to intraoperative bleeding increases for tumors greater than 6 cm [3, 6]. In the described case, where adherence to surrounding structures and obscurement of blood supply were felt to be problematic, laparoscopy was not attempted. Even so, extremes of blood pressure and surgical difficulty were encountered which likely altered blood flow dynamics to the already compromised optic chiasm intraoperatively and brought to light the diagnosis of pituitary adenoma. This prompted the workup for MEN and ultimately the diagnosis of the patient's most mortal disease—his thyroid cancer.

Pheochromocytoma are most commonly sporadic, however 10–23% are associated with a familial syndrome. Workup with CT and/or MRI is recommended for staging and localization purposes. Scintigraphy using ^131^I-MIBG (metaiodobenzylguanidine) is useful to determine the location of occult lesions when CT or MRI are unproductive. There are currently no formal recommendations for workup of associated malignancies when there is no family history. Only 5% of patients with pheochromocytoma present without symptoms, however in patients with MEN-2a, 50% will be asymptomatic [7]. This may be a characteristic of the disease or it may reflect the impact of screening in this population. More investigation may be warranted to determine if patients with incidentally discovered pheochromocytoma warrant workup for a familial cause.

The RET proto-oncogene is a 21 exon gene encoding a tyrosine kinase receptor that is involved in transduction of growth and differentiation signals in developing tissues—particularly those of neural crest origin. RET is strongly associated with MEN-2a and patients with a RET mutation have a nearly 100% chance of developing MTC. Different point mutations in this gene are associated with varying penetrance of pheochromocytoma and hyperparathyroidism, lower age at onset of disease, and likelihood of metastatic disease at presentation. The mutation in the presented patient, *C618S*, indicates substitution of serine for cysteine at point 618 located on exon 10 of the RET gene. This mutation is associated with a high risk of progression and metastasis from MTC [8]. Patients screened for this mutation at birth would typically have a prophylactic thyroidectomy by age of 5 years. Interestingly, this gene is uncommonly associated with pheochromocytoma [8].

Pituitary adenoma is commonly associated with Multiple Endocrine Neoplasia type 1 (MEN 1), however is not associated with MEN-2a. In a recent review, Breckenridge et al. cited 25 cases of patients with pheochromocytoma and pituitary macroadenoma from 1964 to 2003 [9]. It is not certain whether this represents a true association or if these are merely coincidental. Both pheochromocytoma and pituitary adenoma are believed to arise from neural crest cells. Schimke's review of different associated tumors favors sporadic occurrences over a true association [10]. Although in the former series, most tumors were functional—including several presenting with acromegaly, in our patient this was a nonfunctional pituitary macroadenoma. 

More recently, attention has been given to the entity of familial isolated pituitary adenoma (FIPA). Approximately 5% of pituitary adenomas are hereditary in nature but not associated with MEN 1 or Carney complex [11]. A recent series evaluated patients with familial pituitary adenoma and screened for RET mutations and none were found [12]. Mutations of the gene encoding aryl hydrocarbon receptor-interacting protein (AIP) are found in 20% of patients with familial pituitary adenoma [13]. AIP has been shown to have in vivo interaction with RET, though the precise role of this interaction is not completely characterized [12]. Vargiolu et al. evaluated tissue from 28 nonfamilial pituitary adenomas for mutation of either AIP or RET and none were identified [14].

## 4. Conclusion

Overlap of pituitary adenoma with MEN-2a is a rare occurrence and it is unknown to be either sporadic or in association with another syndrome. An interplay between RET proto-oncogene and familial isolated pituitary adenoma may be involved. Asymptomatic pheochromocytoma has a high association with familial syndrome and may warrant further scrutiny. This case represents a truly unique presentation of this very rare phenomenon.

## References

[1] Akerstrom G, Stalberg P (2009). Surgical management of MEN-1 and -2: state of the art. *Surgical Clinics of North America*.

[2] Raue F, Kraimps JL, Dralle H (1995). Primary hyperparathyroidism in multiple endocrine neoplasia type 2A. *Journal of Internal Medicine*.

[3] Humphrey R, Gray D, Pautler S, Davies W (2008). Laparoscopic compared with open adrenalectomy for resection of pheochromocytoma: a review of 47 cases. *Canadian Journal of Surgery*.

[4] Kaye DR, Storey BB, Pacak K, Pinto PA, Linehan WM, Bratslavsky G (2010). Partial adrenalectomy: underused first line therapy for small adrenal tumors. *Journal of Urology*.

[5] Scholten A (2010). Evolution of surgical treatment of primary hyperparathyroidism in multiple endocrine neoplasia type 2A patients. *Endocrine Practice*.

[6] Wilhelm SM, Prinz RA, Barbu AM, Onders RP, Solorzano CC (2006). Analysis of large versus small pheochromocytomas: operative approaches and patient outcomes. *Surgery*.

[7] Pomares FJ, Cañas R, Rodriguez JM, Hernandez AM, Parrilla P, Tebar FJ (1998). Differences between sporadic and multiple endocrine neoplasia type 2A phaeochromocytoma. *Clinical Endocrinology*.

[8] Raue F, Frank-Raue K (2009). Genotype-phenotype relationship in multiple endocrine neoplasia type 2. Implications for clinical management. *Hormones*.

[9] Breckenridge SM, Hamrahian AH, Faiman C, Suh J, Prayson R, Mayberg M (2003). Coexistence of a pituitary macroadenoma and pheochromocytoma—a case report and review of the literature. *Pituitary*.

[10] Schimke RN (1990). Multiple endocrine neoplasia: how many syndromes?. *American Journal of Medical Genetics*.

[11] Daly AF, Tichomirowa MA, Beckers A (2009). Update on familial pituitary tumors: from multiple endocrine neoplasia type 1 to familial isolated pituitary adenoma. *Hormone Research*.

[12] Heliövaara E, Tuupanen S, Ahlsten M (2011). No evidence of RET germline mutations in familial pituitary adenoma. *Journal of Molecular Endocrinology*.

[13] Tahir A, Chahal HS, Korbonits M (2010). Molecular genetics of the aip gene in familial pituitary tumorigenesis. *Progress in Brain Research*.

[14] Vargiolu M, Fusco D, Kurelac I (2009). The tyrosine kinase receptor RET interacts in vivo with aryl hydrocarbon receptor-interacting protein to alter survivin availability. *Journal of Clinical Endocrinology and Metabolism*.

